# Crystal structure of 1,3-bis­[(*E*)-4-meth­oxy­benzyl­idene­amino]­propan-2-ol

**DOI:** 10.1107/S2056989016016947

**Published:** 2016-11-04

**Authors:** Augusto Rivera, Ingrid Miranda-Carvajal, Jaime Ríos-Motta, Michael Bolte

**Affiliations:** a Universidad Nacional de Colombia, Sede Bogotá, Facultad de Ciencias, Departamento de Química, Cra 30 No. 45-03, Bogotá, Código Postal 111321, Colombia; b Institut für Anorganische Chemie, J. W. Goethe-Universität Frankfurt, Max-von Laue-Strasse 7, 60438 Frankfurt/Main, Germany

**Keywords:** crystal structure, hydrogen bonding, C—H⋯O inter­actions, Schiff bases

## Abstract

The title Schiff base, C_19_H_22_N_2_O_3_, was synthesized *via* the condensation reaction of 1,3-di­amino­propan-2-ol with 4-meth­oxy­benzaldehyde using water as solvent. The mol­ecule exists in an *E*,*E* conformation with respect to the C=N imine bonds and the dihedral angle between the aromatic rings is 37.25 (15)°. In the crystal, O—H⋯N hydrogen bonds link the mol­ecules into infinite *C*(5) chains propagating along the *a*-axis direction. The packing of these chains is consolidated by C—H⋯O inter­actions and C—H⋯π short contacts, forming a three-dimensional network.

## Abstract 2

In the crystal of 1,3-bis­[(*E*)-4-meth­oxy­benzyl­idene­amino]­propan-2-ol, mol­ecules are linked by O—H⋯N hydrogen bonds into *C*(5) supra­molecular chains propagating along the *a*-axis direction.

## Chemical context   

Compounds containing the –C=N– (azomethine group) structure are known as Schiff bases, usually synthesized from the condensation of primary amines and active carbonyl groups (Bekdemir & Efil, 2014 ▸). Imines are one class of the most important and fundamental unsaturated organic compounds with a C=N double bond as their characteristic chemical bond and are extensively present in natural products and many drugs (Zhu *et al.*, 2010 ▸). The formation of imines underlies a discipline known as dynamic covalent chemistry (DCC), which is now used widely in the construction of exotic mol­ecules and extended structures such as rotaxanes, caten­anes, and so on (Patil & Adimurthy, 2013 ▸). Schiff base compounds derived from 1,*n*-di­amines play an important role in coordination chemistry and have been studied extensively for their broad range of biological activities (Sahu *et al.* 2012 ▸; da Silva *et al.*, 2011 ▸; Przybylski *et al.* 2009 ▸; Dhar & Taploo, 1982 ▸). The common structural feature of these compounds is the presence of two azomethine groups linked by an *n*-methyl­ene bridge, which can act as hydrogen-bond acceptors.
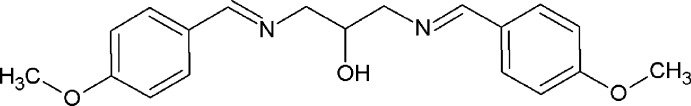


The title compound is inter­esting in that the presence of an OH group in the 1,3-di­amine mol­ecule is situated in a favorable position towards the azomethine groups to form an intra­molecular hydrogen bond. Thus, one may expect that the charge distribution around the azomethine nitro­gen atoms may be visibly perturbed by the presence of this type of inter­action. Hence, 1,3-di­amine­propan-2ol was chosen with the expectation that the presence of an OH group would result in an intra­molecular hydrogen bond. The title compound was synthesized quickly and efficiently by condensation of 1,3-di­amine-2-propanol and *p*-meth­oxy­benzaldehyde using a simple water-mediated procedure that requires neither a catalyst nor any additive (Rivera, *et al.*, 2016 ▸). To the best of our knowledge, no X-ray crystal structure of either the uncoord­inated or the coordinated title compound has been reported previously.

## Structural commentary   

**Figure 1:**
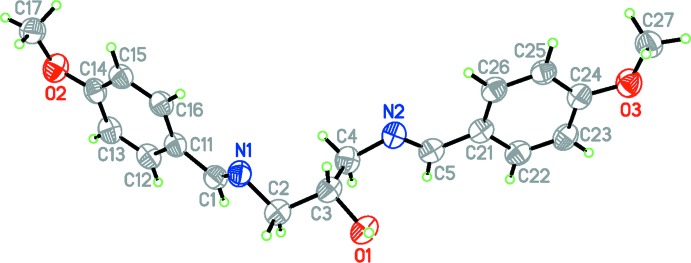
The mol­ecular structure of the title compound, Displacement ellipsoids are drawn at the 50% probability level.

The mol­ecular structure of the title compound is illustrated in Fig. 1 ▸. The title compound exists in an *E,E* conformation with respect to the N1=C1 and N2=C5 azomethine bonds and the C2—N1=C1—C11 and C4—N2=C5—C21 torsion angles are 175.6 (3) and −178.3 (3)° respectively. The N1=C1 and N2=C5 distances of 1.265 (4) and 1.271 (4) Å, respectively, are consistent with C=N double bonding. The bond angles of 117.0 (3) and 117.7 (3)° around the N1 and N2 atoms confirm their *sp* ^2^ character. The slight differences between N=C distances and C—N=C angles are due to the significant effect of the hydrogen bond on the geometric parameters of the nitro­gen atom (N2) involved in the inter­molecular hydrogen bond (see below).

The N1—C2—C3—C4 torsion angle is −64.4 (4)° and the C2—C3—C4—N2 torsion angle is 175.0 (3)°. The O1—C3—C4—N2 torsion angle is −65.3 (3)°, which has a significant role to play in the hydrogen-bonding pattern in the crystal of the title compound (see below). The two meth­oxy substituents are essentially coplanar with their bound benzene rings with torsion angles C17—O2—C14—C13 = 169.3 (3)° and C27—O3—C24—C23 = −172.2 (3)°.

## Supra­molecular features   

**Figure 2:**
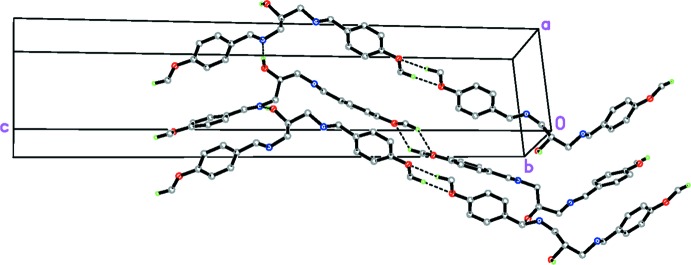
Partial packing diagram of the title compound, showing an extended hydrogen-bonded network. H atoms not involved in hydrogen bonds have been omitted for clarity.

Rather than the proposed intra­molecular O—H⋯N hydrogen bond, adjacent mol­ecules in the crystal of the title compound are linked by inter­molecular O—H⋯N hydrogen bonds (Table 1 ▸, Fig. 2 ▸), forming an infinite zigzag *C*(5) chains extending along the *a*-axis direction. The chains are further linked to neighbouring chains through a pair of weak C—H⋯O hydrogen bonds (Table 1 ▸). Furthermore, C12—H12 and C22—H22 form weaker C—H⋯*Cg* (π–ring) inter­actions (Table 1 ▸), which connect the chains of consecutive layers, thus forming a three-dimensional supra­molecular network (Fig. 2 ▸).

## Database survey   

No comparable structure of either the uncoordinated or the coordinated title compound has been found in the Cambridge Crystallographic Database.

## Synthesis and crystallization   

The title compound was prepared according to our published method (Rivera *et al.*, 2016 ▸). The crude product was dissolved in benzene and aceto­nitrile was added to the solution: upon slow evaporation of the solvent, colorless plates of the title compound arose. M.p. 403 K, yield 88%.

## Refinement   

Crystal data, data collection and structure refinement details are summarized in Table 2 ▸. The hydroxyl H atom was refined freely; however, the remaining H atoms were positioned geometrically and allowed to ride on their parent atoms, with *d*(C—H) = 0.95 Å for aromatic and azomethine atoms, *d*(C—H) = 0.99 Å for methyl­ene, *d*(C—H) = 1.00 Å for C3—H3 and 0.98 Å for CH_3_ atoms. The *U*_iso_ values were constrained to be 1.5*U*_eq_ of the carrier atom for methyl H atoms and 1.2*U*_eq_ for the remaining H atoms. A rotating group model was used for the methyl groups. The absolute structure of the crystal chosen for data collection was indeterminate in the present refinement.

## Supplementary Material

Crystal structure: contains datablock(s) I. DOI: 10.1107/S2056989016016947/hb7623sup1.cif

Structure factors: contains datablock(s) I. DOI: 10.1107/S2056989016016947/hb7623Isup2.hkl

Supporting information file. DOI: 10.1107/S2056989016016947/hb7623Isup3.cml

CCDC reference: 1511139

Additional supporting information: crystallographic information; 3D view; checkCIF report

## Figures and Tables

**Table 1:** Hydrogen-bond geometry (Å, °) *Cg*1 and *Cg*2 are the centroids of the C11–C16 and C21–C26 rings, respectively.

*D*—H⋯*A*	*D*—H	H⋯*A*	*D*⋯*A*	*D*—H⋯*A*
O1—H1⋯N2^i^	0.96 (5)	1.80 (5)	2.761 (4)	175 (4)
C17—H17*B*⋯O3^ii^	0.98	2.63	3.447 (5)	142
C27—H27*B*⋯O2^iii^	0.98	2.65	3.415 (4)	135
C12—H12⋯*Cg*1^iv^	0.95	2.99	3.658 (4)	129
C22—H22⋯*Cg*2^iv^	0.95	2.92	3.628 (3)	132

Symmetry codes: (i) 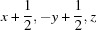; (ii) 
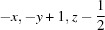
; (iii) 
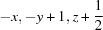
; (iv) 
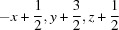
.

**Table 2:** Experimental details

Crystal data	
Chemical formula	C_19_H_22_N_2_O_3_
*M*_r_	326.38
Crystal system, space group	Orthorhombic, *P* *n* *a*2_1_
Temperature (K)	173
*a*, *b*, *c* (Å)	7.9081 (3), 5.8434 (3), 37.4435 (16)
*V* (Å^3^)	1730.27 (13)
*Z*	4
Radiation type	Mo *K*α
μ (mm^−1^)	0.09
Crystal size (mm)	0.28 × 0.19 × 0.08

Data collection	
Diffractometer	Stoe *IPDS* II two-circle
Absorption correction	Multi-scan (*X-AREA*; Stoe & Cie, 2001 ▸)
*T*_min_, *T*_max_	0.625, 1.000
No. of measured, independent and observed [*I* > 2σ(*I*)] reflections	12316, 3084, 2786
*R*_int_	0.038
(sin θ/λ)_max_ (Å^−1^)	0.608

Refinement	
*R*[*F* ^2^ > 2σ(*F* ^2^)], *wR*(*F* ^2^), *S*	0.046, 0.123, 1.01
No. of reflections	3084
No. of parameters	224
No. of restraints	1
H-atom treatment	H atoms treated by a mixture of independent and constrained refinement
Δρ_max_, Δρ_min_ (e Å^−3^)	0.15, −0.16

Computer programs: *X-AREA* (Stoe & Cie, 2001 ▸), *SHELXS97* (Sheldrick, 2008 ▸), *SHELXL2014* (Sheldrick, 2015 ▸) and *XP* in *SHELXTL-Plus* (Sheldrick, 2008 ▸).

## supplementary crystallographic information

### Crystal data

**Table:**

C_19_H_22_N_2_O_3_	*D*_x_ = 1.253 Mg m^−^^3^
*M**_r_* = 326.38	Mo *K*α radiation, λ = 0.71073 Å
Orthorhombic, *P**n**a*2_1_	Cell parameters from 12316 reflections
*a* = 7.9081 (3) Å	θ = 2.2–26.0°
*b* = 5.8434 (3) Å	µ = 0.09 mm^−^^1^
*c* = 37.4435 (16) Å	*T* = 173 K
*V* = 1730.27 (13) Å^3^	Plate, colourless
*Z* = 4	0.28 × 0.19 × 0.08 mm
*F*(000) = 696	

### Data collection

**Table:**

Stoe IPDS II two-circle diffractometer	2786 reflections with *I* > 2σ(*I*)
Radiation source: Genix 3D IµS microfocus X-ray source	*R*_int_ = 0.038
ω scans	θ_max_ = 25.6°, θ_min_ = 2.2°
Absorption correction: multi-scan (X-AREA; Stoe & Cie, 2001)	*h* = −9→9
*T*_min_ = 0.625, *T*_max_ = 1.000	*k* = −7→7
12316 measured reflections	*l* = −41→45
3084 independent reflections	

### Refinement

**Table:**

Refinement on *F*^2^	Hydrogen site location: mixed
Least-squares matrix: full	H atoms treated by a mixture of independent and constrained refinement
*R*[*F*^2^ > 2σ(*F*^2^)] = 0.046	*w* = 1/[σ^2^(*F*_o_^2^) + (0.0961*P*)^2^] where *P* = (*F*_o_^2^ + 2*F*_c_^2^)/3
*wR*(*F*^2^) = 0.123	(Δ/σ)_max_ < 0.001
*S* = 1.01	Δρ_max_ = 0.15 e Å^−^^3^
3084 reflections	Δρ_min_ = −0.16 e Å^−^^3^
224 parameters	Extinction correction: SHELXL2014 (Sheldrick, 2015), Fc^*^=kFc[1+0.001xFc^2^λ^3^/sin(2θ)]^-1/4^
1 restraint	Extinction coefficient: 0.046 (8)

### Special details

**Table:**

Geometry. All esds (except the esd in the dihedral angle between two l.s. planes) are estimated using the full covariance matrix. The cell esds are taken into account individually in the estimation of esds in distances, angles and torsion angles; correlations between esds in cell parameters are only used when they are defined by crystal symmetry. An approximate (isotropic) treatment of cell esds is used for estimating esds involving l.s. planes.

### Fractional atomic coordinates and isotropic or equivalent isotropic displacement parameters (Å^2^)

**Table:**

	*x*	*y*	*z*	*U*_iso_*/*U*_eq_	
N1	0.5510 (4)	0.4188 (4)	0.44173 (8)	0.0467 (6)	
N2	0.3474 (4)	0.4764 (4)	0.54792 (8)	0.0453 (6)	
O1	0.7066 (3)	0.4483 (4)	0.53519 (7)	0.0513 (6)	
H1	0.754 (7)	0.302 (9)	0.5410 (13)	0.073 (13)*	
O2	0.1888 (4)	0.4687 (4)	0.28786 (6)	0.0547 (6)	
O3	0.0967 (3)	0.4509 (4)	0.71286 (7)	0.0542 (6)	
C1	0.5258 (4)	0.5772 (6)	0.41931 (9)	0.0463 (7)	
H1A	0.5738	0.7234	0.4238	0.056*	
C2	0.6638 (5)	0.4706 (6)	0.47176 (9)	0.0518 (8)	
H2A	0.6906	0.6361	0.4716	0.062*	
H2B	0.7711	0.3856	0.4687	0.062*	
C3	0.5858 (4)	0.4072 (5)	0.50766 (9)	0.0461 (7)	
H3	0.5540	0.2416	0.5076	0.055*	
C4	0.4306 (4)	0.5507 (5)	0.51505 (9)	0.0491 (8)	
H4A	0.4637	0.7134	0.5173	0.059*	
H4B	0.3507	0.5371	0.4948	0.059*	
C5	0.3529 (4)	0.6102 (5)	0.57466 (9)	0.0448 (7)	
H5	0.4082	0.7533	0.5717	0.054*	
C11	0.4253 (4)	0.5452 (5)	0.38653 (9)	0.0414 (7)	
C12	0.4251 (5)	0.7189 (5)	0.36069 (10)	0.0477 (7)	
H12	0.4806	0.8597	0.3655	0.057*	
C13	0.3447 (4)	0.6874 (6)	0.32820 (9)	0.0484 (8)	
H13	0.3464	0.8057	0.3108	0.058*	
C14	0.2617 (4)	0.4832 (5)	0.32098 (9)	0.0442 (7)	
C15	0.2578 (4)	0.3105 (5)	0.34676 (9)	0.0436 (7)	
H15	0.1994	0.1716	0.3421	0.052*	
C16	0.3392 (4)	0.3430 (5)	0.37899 (8)	0.0437 (7)	
H16	0.3365	0.2247	0.3964	0.052*	
C17	0.1278 (5)	0.2506 (6)	0.27655 (10)	0.0553 (8)	
H17A	0.2219	0.1415	0.2758	0.083*	
H17B	0.0778	0.2645	0.2527	0.083*	
H17C	0.0420	0.1961	0.2934	0.083*	
C21	0.2798 (4)	0.5588 (5)	0.60975 (9)	0.0435 (7)	
C22	0.3029 (4)	0.7163 (5)	0.63743 (9)	0.0472 (7)	
H22	0.3628	0.8540	0.6328	0.057*	
C23	0.2409 (5)	0.6766 (5)	0.67121 (9)	0.0499 (8)	
H23	0.2581	0.7865	0.6896	0.060*	
C24	0.1526 (4)	0.4751 (5)	0.67851 (9)	0.0445 (7)	
C25	0.1277 (4)	0.3152 (5)	0.65135 (9)	0.0445 (7)	
H25	0.0677	0.1776	0.6561	0.053*	
C26	0.1910 (4)	0.3579 (5)	0.61733 (9)	0.0455 (7)	
H26	0.1734	0.2485	0.5989	0.055*	
C27	0.0252 (5)	0.2384 (6)	0.72315 (10)	0.0582 (9)	
H27A	−0.0747	0.2069	0.7085	0.087*	
H27B	−0.0079	0.2450	0.7484	0.087*	
H27C	0.1086	0.1163	0.7197	0.087*	

### Atomic displacement parameters (Å^2^)

**Table:**

	*U*^11^	*U*^22^	*U*^33^	*U*^12^	*U*^13^	*U*^23^
N1	0.0514 (14)	0.0482 (13)	0.0404 (13)	−0.0007 (12)	0.0008 (12)	−0.0031 (12)
N2	0.0506 (15)	0.0425 (13)	0.0429 (14)	0.0044 (11)	−0.0030 (12)	−0.0012 (11)
O1	0.0585 (14)	0.0457 (12)	0.0498 (13)	0.0004 (11)	−0.0133 (11)	−0.0031 (10)
O2	0.0675 (16)	0.0507 (13)	0.0459 (14)	0.0031 (11)	−0.0074 (11)	0.0050 (10)
O3	0.0623 (15)	0.0522 (13)	0.0479 (13)	−0.0001 (11)	0.0034 (12)	−0.0082 (10)
C1	0.0470 (17)	0.0461 (16)	0.0459 (18)	−0.0049 (14)	0.0055 (14)	−0.0027 (13)
C2	0.0499 (18)	0.056 (2)	0.049 (2)	−0.0019 (14)	−0.0035 (15)	−0.0018 (14)
C3	0.0507 (17)	0.0425 (15)	0.0451 (17)	−0.0028 (13)	−0.0076 (14)	−0.0013 (13)
C4	0.0588 (18)	0.0434 (16)	0.0451 (18)	0.0032 (14)	−0.0021 (14)	0.0040 (14)
C5	0.0472 (17)	0.0373 (15)	0.0501 (18)	0.0021 (12)	−0.0068 (14)	0.0020 (13)
C11	0.0440 (16)	0.0396 (16)	0.0405 (16)	0.0009 (12)	0.0048 (13)	−0.0003 (12)
C12	0.0526 (18)	0.0377 (15)	0.0529 (18)	0.0008 (14)	0.0095 (14)	0.0011 (13)
C13	0.0595 (19)	0.0396 (16)	0.0462 (19)	0.0048 (13)	0.0062 (14)	0.0082 (13)
C14	0.0446 (16)	0.0453 (16)	0.0428 (17)	0.0074 (12)	0.0050 (14)	0.0021 (13)
C15	0.0464 (16)	0.0381 (16)	0.0464 (17)	−0.0004 (13)	0.0050 (13)	0.0016 (12)
C16	0.0482 (17)	0.0396 (15)	0.0432 (17)	0.0010 (13)	0.0043 (13)	0.0059 (13)
C17	0.0550 (19)	0.061 (2)	0.0503 (19)	−0.0044 (16)	−0.0028 (15)	0.0035 (15)
C21	0.0439 (16)	0.0383 (15)	0.0482 (18)	0.0037 (12)	−0.0056 (13)	−0.0018 (12)
C22	0.0511 (18)	0.0375 (16)	0.0530 (18)	−0.0005 (13)	−0.0054 (15)	−0.0027 (13)
C23	0.0587 (19)	0.0419 (15)	0.0491 (19)	0.0014 (15)	−0.0078 (15)	−0.0095 (13)
C24	0.0453 (16)	0.0438 (16)	0.0444 (17)	0.0066 (13)	−0.0016 (13)	−0.0049 (13)
C25	0.0459 (16)	0.0398 (16)	0.0478 (17)	−0.0018 (13)	−0.0018 (14)	−0.0040 (13)
C26	0.0486 (17)	0.0400 (15)	0.0478 (17)	0.0019 (13)	−0.0026 (14)	−0.0082 (13)
C27	0.060 (2)	0.062 (2)	0.0529 (19)	−0.0114 (17)	0.0086 (17)	−0.0079 (15)

### Geometric parameters (Å, º)

**Table:**

N1—C1	1.265 (4)	C12—H12	0.9500
N1—C2	1.467 (5)	C13—C14	1.388 (5)
N2—C5	1.271 (4)	C13—H13	0.9500
N2—C4	1.461 (4)	C14—C15	1.397 (5)
O1—C3	1.426 (4)	C15—C16	1.381 (5)
O1—H1	0.96 (5)	C15—H15	0.9500
O2—C14	1.370 (4)	C16—H16	0.9500
O2—C17	1.427 (4)	C17—H17A	0.9800
O3—C24	1.367 (4)	C17—H17B	0.9800
O3—C27	1.418 (4)	C17—H17C	0.9800
C1—C11	1.474 (5)	C21—C26	1.397 (5)
C1—H1A	0.9500	C21—C22	1.398 (5)
C2—C3	1.525 (5)	C22—C23	1.376 (5)
C2—H2A	0.9900	C22—H22	0.9500
C2—H2B	0.9900	C23—C24	1.396 (5)
C3—C4	1.511 (5)	C23—H23	0.9500
C3—H3	1.0000	C24—C25	1.395 (4)
C4—H4A	0.9900	C25—C26	1.391 (5)
C4—H4B	0.9900	C25—H25	0.9500
C5—C21	1.466 (5)	C26—H26	0.9500
C5—H5	0.9500	C27—H27A	0.9800
C11—C16	1.393 (4)	C27—H27B	0.9800
C11—C12	1.402 (5)	C27—H27C	0.9800
C12—C13	1.385 (5)		
			
C1—N1—C2	117.0 (3)	O2—C14—C15	124.8 (3)
C5—N2—C4	117.7 (3)	C13—C14—C15	119.8 (3)
C3—O1—H1	106 (3)	C16—C15—C14	119.6 (3)
C14—O2—C17	117.8 (3)	C16—C15—H15	120.2
C24—O3—C27	118.4 (3)	C14—C15—H15	120.2
N1—C1—C11	123.0 (3)	C15—C16—C11	121.4 (3)
N1—C1—H1A	118.5	C15—C16—H16	119.3
C11—C1—H1A	118.5	C11—C16—H16	119.3
N1—C2—C3	112.3 (3)	O2—C17—H17A	109.5
N1—C2—H2A	109.1	O2—C17—H17B	109.5
C3—C2—H2A	109.1	H17A—C17—H17B	109.5
N1—C2—H2B	109.1	O2—C17—H17C	109.5
C3—C2—H2B	109.1	H17A—C17—H17C	109.5
H2A—C2—H2B	107.9	H17B—C17—H17C	109.5
O1—C3—C4	108.6 (3)	C26—C21—C22	117.9 (3)
O1—C3—C2	109.0 (3)	C26—C21—C5	123.5 (3)
C4—C3—C2	110.8 (3)	C22—C21—C5	118.5 (3)
O1—C3—H3	109.5	C23—C22—C21	121.6 (3)
C4—C3—H3	109.5	C23—C22—H22	119.2
C2—C3—H3	109.5	C21—C22—H22	119.2
N2—C4—C3	110.8 (3)	C22—C23—C24	120.0 (3)
N2—C4—H4A	109.5	C22—C23—H23	120.0
C3—C4—H4A	109.5	C24—C23—H23	120.0
N2—C4—H4B	109.5	O3—C24—C25	124.8 (3)
C3—C4—H4B	109.5	O3—C24—C23	115.7 (3)
H4A—C4—H4B	108.1	C25—C24—C23	119.5 (3)
N2—C5—C21	124.5 (3)	C26—C25—C24	119.8 (3)
N2—C5—H5	117.8	C26—C25—H25	120.1
C21—C5—H5	117.8	C24—C25—H25	120.1
C16—C11—C12	118.3 (3)	C25—C26—C21	121.2 (3)
C16—C11—C1	122.7 (3)	C25—C26—H26	119.4
C12—C11—C1	118.9 (3)	C21—C26—H26	119.4
C13—C12—C11	120.7 (3)	O3—C27—H27A	109.5
C13—C12—H12	119.7	O3—C27—H27B	109.5
C11—C12—H12	119.7	H27A—C27—H27B	109.5
C12—C13—C14	120.2 (3)	O3—C27—H27C	109.5
C12—C13—H13	119.9	H27A—C27—H27C	109.5
C14—C13—H13	119.9	H27B—C27—H27C	109.5
O2—C14—C13	115.4 (3)		
			
C2—N1—C1—C11	175.6 (3)	C13—C14—C15—C16	−1.2 (4)
C1—N1—C2—C3	129.7 (3)	C14—C15—C16—C11	0.2 (5)
N1—C2—C3—O1	176.2 (3)	C12—C11—C16—C15	1.1 (5)
N1—C2—C3—C4	−64.4 (4)	C1—C11—C16—C15	−174.5 (3)
C5—N2—C4—C3	110.7 (3)	N2—C5—C21—C26	−2.0 (5)
O1—C3—C4—N2	−65.3 (3)	N2—C5—C21—C22	176.6 (3)
C2—C3—C4—N2	175.0 (3)	C26—C21—C22—C23	0.1 (5)
C4—N2—C5—C21	−178.3 (3)	C5—C21—C22—C23	−178.6 (3)
N1—C1—C11—C16	5.1 (5)	C21—C22—C23—C24	0.1 (5)
N1—C1—C11—C12	−170.5 (3)	C27—O3—C24—C25	7.6 (5)
C16—C11—C12—C13	−1.6 (5)	C27—O3—C24—C23	−172.2 (3)
C1—C11—C12—C13	174.3 (3)	C22—C23—C24—O3	179.7 (3)
C11—C12—C13—C14	0.6 (5)	C22—C23—C24—C25	−0.1 (5)
C17—O2—C14—C13	169.3 (3)	O3—C24—C25—C26	−179.8 (3)
C17—O2—C14—C15	−10.5 (5)	C23—C24—C25—C26	0.1 (5)
C12—C13—C14—O2	−179.0 (3)	C24—C25—C26—C21	0.1 (5)
C12—C13—C14—C15	0.8 (5)	C22—C21—C26—C25	−0.2 (5)
O2—C14—C15—C16	178.6 (3)	C5—C21—C26—C25	178.4 (3)

### Hydrogen-bond geometry (Å, º)

Cg1 and Cg2 are the centroids of the C11–C16 and C21–C26 rings, respectively.

**Table:**

*D*—H···*A*	*D*—H	H···*A*	*D*···*A*	*D*—H···*A*
O1—H1···N2^i^	0.96 (5)	1.80 (5)	2.761 (4)	175 (4)
C17—H17*B*···O3^ii^	0.98	2.63	3.447 (5)	142
C27—H27*B*···O2^iii^	0.98	2.65	3.415 (4)	135
C12—H12···*Cg*1^iv^	0.95	2.99	3.658 (4)	129
C22—H22···*Cg*2^iv^	0.95	2.92	3.628 (3)	132

Symmetry codes: (i) *x*+1/2, −*y*+1/2, *z*; (ii) −*x*, −*y*+1, *z*−1/2; (iii) −*x*, −*y*+1, *z*+1/2; (iv) −*x*+1/2, *y*+3/2, *z*+1/2.
